# Virtual Reality for Upper Limb Rehabilitation in Patients With Obstetric Brachial Palsy: Systematic Review and Meta-Analysis of Randomized Controlled Trials

**DOI:** 10.2196/47391

**Published:** 2023-06-30

**Authors:** Amaranta De Miguel-Rubio, Alvaro Alba-Rueda, Elena María Millán-Salguero, M Dolores De Miguel-Rubio, Jose A Moral-Munoz, David Lucena-Anton

**Affiliations:** 1 Departamento de Enfermería, Farmacología y Fisioterapia Universidad de Córdoba Córdoba Spain; 2 Centro Docente Privado Albor Córdoba Spain; 3 Departamento de Enfermería y Fisioterapia, Instituto de Investigación e Innovación Biomédica de Cádiz (INiBICA), Facultad de Enfermería y Fisioterapia Universidad de Cádiz Cádiz Spain

**Keywords:** neonatal brachial plexus palsy, virtual reality, rehabilitation, upper extremity, review, meta-analysis

## Abstract

**Background:**

Obstetric brachial palsy (OBP) is a pathology caused by complications during childbirth because of cervical spine elongation, affecting the motor and sensory innervation of the upper limbs. The most common lesion occurs on the C5 and C6 nerve branches, known as Erb-Duchenne palsy. The least common lesion is when all nerve roots are affected (C5-T1), which has the worst prognosis. Virtual reality (VR) is commonly used in neurological rehabilitation for the evaluation and treatment of physical deficits.

**Objective:**

This systematic review aims to assess the efficacy of VR in the rehabilitation of upper limb function in patients with OBP.

**Methods:**

A search was performed according to the PRISMA (Preferred Reporting Items for Systematic Reviews and Meta-Analyses) 2020 guidelines in several scientific databases—PubMed, Web of Science, PEDro, Cochrane, MEDLINE, Scopus, and CINAHL—without language or date restrictions and including articles published up to April 2023. The inclusion criteria were established according to the population, intervention, comparison, outcome, and study (PICOS) design framework: children aged <18 years diagnosed with OBP, VR therapy used in addition to conventional therapy or isolated, VR therapy compared with conventional therapy, outcomes related to OBP rehabilitation therapy, and randomized controlled trials (RCTs). The PEDro scale was used to assess the methodological quality of the RCTs, and the Cochrane Collaboration tool was used to assess the risk of bias. The Review Manager statistical software (version 5.4; The Cochrane Collaboration) was used to conduct the meta-analysis. The results were synthesized through information extraction and presented in tables and forest plots.

**Results:**

In total, 5 RCTs were included in this systematic review, with 3 (60%) providing information for the meta-analysis. A total of 138 participants were analyzed. All the studies used semi-immersive or nonimmersive VR systems. The statistical analysis showed no favorable results for all outcomes except for the hand-to-mouth subtest of the Mallet scoring system (functional activity; standardized mean difference −0.97, 95% CI −1.67 to −0.27; *P*=.007).

**Conclusions:**

The evidence for the use of VR therapy for upper limb rehabilitation outcomes in patients with OBP was insufficient to support its efficacy and strongly recommend its use. Nevertheless, scientific literature supports the use of VR technologies for rehabilitation as it provides several advantages, such as enhancing the patient’s motivation, providing direct feedback, and focusing the patient’s attention during the intervention. Thus, the use of VR for upper limb rehabilitation in patients with OBP is still in its first stages. Small sample sizes; limited long-term analysis; lack of testing of different doses; and absence of International Classification of Functioning, Disability, and Health–related outcomes were present in the included RCTs, so further research is needed to fully understand the potential of VR technologies as a therapeutic approach for patients with OBP.

**Trial Registration:**

PROSPERO CRD42022314264; https://www.crd.york.ac.uk/prospero/display_record.php?RecordID=314264

## Introduction

### Background

The brachial plexus is the network of nerves that provides motor and sensory innervation to the upper extremities and comprises the anterior branches of the spinal nerves (C5-T1) [1,2]. It can be damaged during delivery through head traction, although it can also occur without it. This elongation results from an increase in the angle between the neck and shoulder, causing the nerves to stretch beyond their capacity. This condition is known as obstetric brachial palsy (OBP), with an incidence of 1.6 to 2.6 per 1000 births. Furthermore, some cases of OBP also occur congenitally because of poor fetal posture, tumors, and uterine issues, with a frequency of 0.3 to 3 per 1000 newborns [3,4]. Newborns most frequently show right upper extremity palsy [1] because of the typical left occiput anterior fetal presentation during delivery and added maneuvers of expulsion causing overstretching of the right shoulder when shoulder dystocia occurs [5-7]. There are other associated risk factors, among them the weight of the newborn (in fetuses whose weight is >4000 g, this complication is 6% more likely to occur) [8,9]. Other influential factors include gestational or pregestational diabetes, macrosomic fetus, pelvic delivery with cervical hyperextension of the neonate, idiopathic factors, and obesity [10]. The classification of brachial plexus involvement can be based on the severity of the injury (preganglionic or avulsion and postganglionic or rupture) or the level of the lesion (proximal, distal, and global) [11,12].

The most common pattern of OBP affects the upper trunk nerves C5 and C6, known as Erb-Duchenne palsy (47%) [13], and has a good prognosis with spontaneous recovery in most cases. Patients usually present with an adducted arm with internal rotation of the shoulder, extended elbow, and flexed wrist. The second pattern involves the upper and middle trunk (C5-C7) and is known as extended Erb-Duchenne palsy. It has a worse prognosis, and the motor manifestations are similar to the previous one, but patients can present with a flexed elbow. Total plexus paralysis affects nerves C5 to C8 and sometimes T1 and is the second most common type of lesion, with the worst prognosis, leading to a clawed hand and a flaccid and insensitive arm [2,5] often accompanied by Horner syndrome (miosis, anhidrosis, and palpebral ptosis). Isolated lesions of C8 and T1, known as Klumpke palsy, are extremely rare (2%) [5] and present with poor grasping of the hand without involvement of the proximal roots of the brachial plexus [11,14]. The functional limitations caused by this condition imply the need to apply specific therapies to facilitate manual skills on the affected side, improving its integration into the child’s natural environment.

Regarding the current standards of care for patients with OBP, on the one hand, initial treatment focuses on physiotherapy based on passive and active mobilizations, stretching, and parent education to prevent contractures of the affected muscles, with follow-up at the age of 3 months to assess biceps function [15,16]. If there is adequate muscle integrity, conservative follow-up and regular monitoring continue until the age of 2 years. However, current conservative management for patients with OBP is not standardized, highlighting the limitations and the need for the development of international consensus clinical guidelines [7]. In contrast, if there is no muscle activity in the first 3 months or if Horner syndrome is present, magnetic resonance imaging should be requested to assess the extent of the lesion or identify neuromas to determine the need for surgical treatment either for grafting or nerve transfer depending on the case, which should be performed before the age of 9 months to prevent permanent damage to the motor plate of the affected muscle [14,17-19].

Virtual reality (VR) can be defined as a computer-generated simulation of a real environment in which, through a human-machine interface, the subject can interact with certain elements within a simulated space [20]. VR systems generate a 3D space in which the user can move freely and interact with stimuli in real time in a computer-generated environment [21]. The term VR includes a large number of technical devices and systems with different characteristics, which can be divided into two groups according to the patient’s level of immersion: (1) immersive systems, where users are fully integrated into the virtual world, including an environment with multisensory input via head-mounted displays, large-screen projection, and VR caves, and (2) semi-immersive or nonimmersive systems, where a computer screen displays the environment, such as video game consoles, which do not require high-quality graphics or special hardware, hence its low cost and accessibility for treatment [22].

Among the different current treatments introduced in the field of rehabilitation, VR allows users to interact with a multitude of environments and scenarios in real time, carrying out intensive, repetitive, and task-focused training such as picking up a personal hygiene utensil [23,24]. Being a game-based therapy, it is a motivating tool that promotes the intervention and adherence of the participants (and their families) in the treatment as, sometimes, conventional therapies may seem boring and monotonous, especially for people of younger ages, as is the case of patients with OBP [25]. Thus, while playing, children acquire experiences that they can apply to their daily lives, and by actively participating, they work on motor skills more effectively [26]. In addition, VR has great advantages, being used as a treatment for different pathologies such as stroke, in which a higher activity rate and longer time of use were demonstrated [27]. In addition, some modalities can be performed with minimal supervision of the physiotherapist, which allows users to increase the treatment time focused on the upper limbs as the main objective after stroke is usually to achieve the patient’s gait [28]. Moreover, VR also shows advantages in phantom limb treatment over other therapies as it can be adapted to the personal characteristics of the patient, such as the patient’s perception of the amputated limb [29]. Nonetheless, there are some challenges that should be considered. In terms of implementing VR-based interventions in clinical practice, it is important to acknowledge that patients may react differently because of their ability to learn in a virtual setting, their sensitivity, or their apprehension. In addition, there may be potential side effects such as cybersickness that can occur during the intervention. Another challenge is that clinicians need specific training to properly use VR technologies for therapy [30]. Finally, the cost of integrating this therapy into clinical practice may be high because of the need to purchase high-quality hardware and software [31].

### Objectives

On the basis of this background, we hypothesized that a systematic review and meta-analysis of randomized controlled trials (RCTs) would provide sufficient scientific evidence to consider VR an effective therapy for upper limb rehabilitation in patients with OBP. Therefore, the overall objective was to assess the efficacy of using VR as a rehabilitation therapy for the affected upper limb in patients with OBP. As specific objectives, we aimed to identify the VR devices used in the recovery of the affected upper limb in patients with OBP and assess the efficacy of VR therapy on functional activity, strength, and range of movement (ROM) in patients with OBP.

## Methods

This systematic review was carried out following the PRISMA (Preferred Reporting Items for Systematic Reviews and Meta-Analyses) recommendations (Multimedia Appendix 1 [32]). In addition, this review was registered in the PROSPERO database (CRD42022314264).

### Search Strategy

A literature search was performed up to April 2023 in the following scientific literature databases: PubMed, Web of Science, PEDro, Cochrane, MEDLINE, Scopus, and CINAHL Complete. In addition, the bibliographic references of the selected articles were reviewed with the intention of finding other studies that could also be included. No filters regarding language or publication date were applied. The search strategy was first developed for the PubMed database and was adapted for the remaining databases using the following keywords and Medical Subject Headings (MeSH) descriptors: “Neonatal Brachial Plexus Palsy” (MeSH), “Brachial Plexus Neuropathies” (MeSH), “Brachial Plexus” (MeSH), “brachial plexus,” “brachial plexus block*,” “brachial plexus blockade*,” “brachial plexus neuritis,” “brachial neuritis,” “amyotrophic neuralgia,” “neurologic amyotrophy,” “brachial neuralgia,” “cervicobrachial neuralgia,” “brachial plexopathy,” “brachial plexus neuropathy,” “brachial plexus neuropathies,” “brachial plexus disorder*,” “brachial plexus disease*,” “klumpke paralysis,” “erb paralysis,” “klumpke palsy,” “erb palsy,” “neonatal brachial plexus palsy,” “obstetrical brachial plexus palsy,” “obstetrical brachial plexus lesion,” “Virtual Reality” (MeSH), “Virtual Reality Exposure Therapy” (MeSH), “Exergaming” (MeSH), “virtual reality exposure therapy,” “virtual reality,” “augmented reality,” “virtual system*,” “video game*,” “videogame*,” “exergaming,” “exergame*,” “commercial game*,” “play-based,” and “game-based.” The Boolean descriptors “AND” and “OR” were used to create the specific search strategy, which is shown in Multimedia Appendix 2.

### Eligibility Criteria

The population, intervention, comparison, outcome, and study (PICOS) design framework [33] was used to define the inclusion criteria: (1) children aged <18 years diagnosed with OBP (population); (2) VR therapy used in addition to conventional therapy or isolated (intervention); (3) conventional therapy (comparison); (4) outcome related to OBP rehabilitation, such as active ROM, functional activity, muscle strength, fine motor skills, or quality of life (outcome); and (5) RCTs (study design).

Studies were excluded from this review if (1) patients had other pathologies in addition to OBP without separately detailing the results between populations and (2) the articles were published in abstract form.

### Screening and Selection Process

In total, 2 reviewers (ADM-R and AAR) independently conducted the search using the search strategy described in Multimedia Appendix 2 and performed the full screening and selection process according to the PRISMA guidelines. A third reviewer (DLA) was involved in resolving conflicts during the process. The screening and selection process carried out by the 2 independent reviewers included the following steps: (1) initial screening to remove duplicates, (2) revision of the titles and abstracts of the studies and exclusion of those whose topic did not fall within the scope of this systematic review, and (3) thorough revision of the full texts of the remaining studies to filter them according to the established inclusion and exclusion criteria. The RCTs that passed all these filters were included in the systematic review.

### Data Extraction Process

The following data were extracted from each article: author, year, age and sex of the sample, OBP lesion level as well as the affected arm for most of the patients in each study, cognitive abilities, type of intervention, session and intervention duration, study outcomes, measurement instruments, and the main results obtained. Data extraction was performed according to the PRISMA guidelines by 2 independent reviewers (ADM-R and AAR). A third reviewer (DLA) participated in resolving conflicts during the process.

### Assessment of the Methodological Quality and Risk of Bias of the Included Studies

The PEDro scale [34] was used to assess the methodological quality of the studies. Owing to the different characteristics of the RCTs included in this study, which involved interventions that made it difficult to conduct double-blind trials, the PEDro scale is a suitable tool commonly used in systematic reviews analyzing the methodological quality of these types of interventions [35]. This scale consists of 11 items related to the domains of selection, performance, detection, information, and attribution. Each item is scored with 1 point if the study meets the criteria except for criterion 1. A higher score indicates a higher methodological quality. A study with a PEDro score of ≥6 is considered to have a high level of methodological quality (6-8: good; 9-10: excellent), and a study with a score of ≤5 is considered to have a low level of methodological quality (4-5: fair; <4: poor) [36].

The risk of bias of the included RCTs was assessed following the recommendations of the Cochrane Collaboration [37] using the Review Manager software (version 5.4; The Cochrane Collaboration). This software includes a description and assessment for each item, including the response to a question where “yes” indicates a low risk of bias, “no” indicates a high risk of bias, and “unclear” indicates a lack of information or uncertainty about the possible bias.

The methodological quality and risk-of-bias assessments were performed by 2 independent authors (ADM-R and AAR), and a third reviewer (DLA) participated in resolving conflicts during the process.

### Statistical Analysis

The meta-analysis was carried out through the Review Manager software. First, the mean differences and SDs of each study group were obtained considering postintervention and baseline values to show intragroup changes. The standardized mean difference (SMD) was used to quantify the effect size of intergroup change differences using a 95% CI. When studies reported the median and IQR, these values were converted to mean and SD using (1) mean=median and (2) SD=IQR/1.35 [38]. The inverse variance method with continuous variables was used. Fixed-effects (*P*≥.05) or random-effects (*P*<.05) models were used according to heterogeneity (using the *I*^2^ statistic and the chi-square test). *I*^2^ values between 0% and 40% were considered not important, between 30% and 60% represented moderate heterogeneity, and between 75% and 100% represented considerable heterogeneity [39]. The RCTs were stratified into different meta-analysis groups according to the outcome measured.

## Results

### Overview

**Figure 1:**
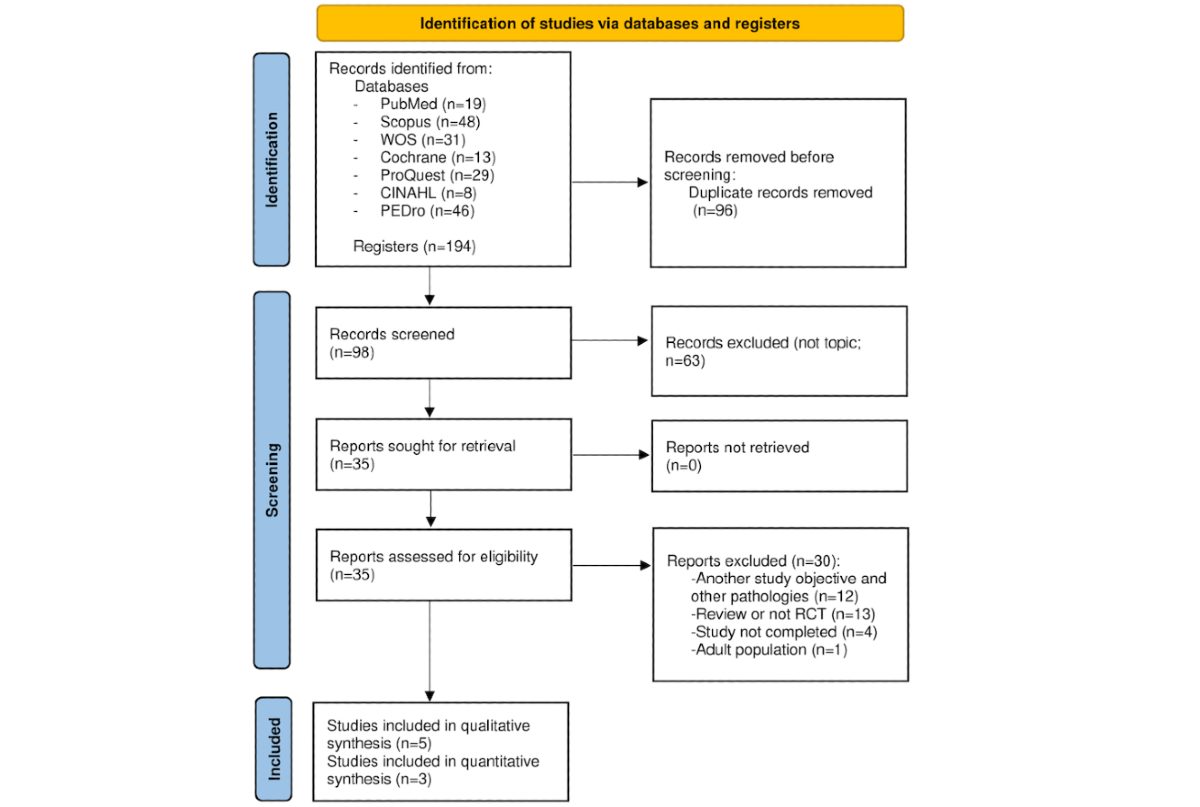
Information flow diagram of the systematic review selection process following the PRISMA (Preferred Reporting Items for Systematic Reviews and Meta-Analyses) recommendations [32]. RCT: randomized controlled trial; WOS: Web of Science.

The literature search retrieved a total of 194 articles, of which 96 (49.5%) were duplicates. From the 98 remaining articles, those that were not related to our search objectives were subtracted, resulting in 35 (36%) articles. Finally, after verifying strict compliance with the inclusion criteria, 5 RCTs were included in this systematic review, as shown in Figure 1.

### Description of the Studies

Concerning the RCTs included in the qualitative synthesis, a total of 138 participants (control group: n=68, 49.3%; experimental group or VR group: n=70, 50.7%), 75 (54.3%) men and 63 (45.7%) women, with OBP were involved. The study by Alsakhawi and Atya [40] had the highest number of participants (N=45), and the study by Yeves-Lite et al [11] had the smallest sample size (N=12). The mean age of the participants ranged from 6.1 [40] to 8.5 years [11]. Regarding the nerve lesion level, all the RCTs (5/5, 100%) [11,40-43] included participants with C5 and C6 nerve lesions, whereas participants with C5 to C7 [11,40,43], C6 and C7 [40], and C5 to T1 [43] nerve lesions were distributed across the different studies.

This systematic review and meta-analysis analyzed the effects of VR compared with conventional therapies on patients diagnosed with OBP. VR therapy was provided through different devices, such as the Armeo Spring Pediatric system (a tool that combines robotic assistance and VR to provide an engaging environment to achieve the required repetitive practice that the upper extremity needs for improved function) [41], E-LINK Upper Limb Exerciser (a device with electronic equipment for active and resistive exercises of the upper limbs, isometric pinch, and grip strength exercise) [40], Nintendo Wii (Nintendo Wii Sports video game console) [42], Leap Motion Controller (a low-cost and low-complexity optoelectronic system that can track hand and finger movements with accuracy) [43], and VR mirror therapy (a mobile app simulating mirror therapy) [11]. Conversely, the control groups included conventional physical therapy (functional, daily living, and game-based activities) [40-43] and conventional mirror therapy (through a mirror, the unaffected upper limb is visualized as the affected one) [11].

El-Shamy and Alsharif [41] had the longest total duration of the intervention and the highest intensity (3 times/week for 12 weeks). In addition, Tarakci et al [43] and Alsakhawi and Atya [40] had the longest session duration (60 minutes). Conversely, Yeves-Lite et al [11] had the shortest intervention time and lowest intensity, only performing 12 sessions (3 times/week for 4 weeks) of 20 minutes.

The RCTs used various assessment tools to evaluate different outcomes of the participants. Functional activity was measured using the Mallet scoring system (MSS) [40-42], Duruoz Hand Index [11], Jebsen-Taylor Hand Function Test [11], Childhood Health Assessment Questionnaire [11], Nine-Hole Peg Test [43], and Children’s Hand-use Experience Questionnaire [11]. Muscle strength was assessed using the Active Movement Scale (AMS) [40,42], a handheld dynamometer [41,43], and a hydraulic pinch gauge [43]. ROM was assessed using a goniometer [41,42] or inclinometer [40]. Finally, quality of life was assessed using the Pediatric Quality of Life Inventory Generic Core Scale [11].

All the RCTs (5/5, 100%) [11,40-43] used parametric (independent or paired *t* test) or nonparametric (Mann-Whitney *U* test and Wilcoxon test) analyses to determine the intergroup differences in the outcomes and compare the before-and-after treatment differences in the same group for both therapies. The level of significance was set at .05. In addition, the effect size was measured in the study by Tarakci et al [43].

The detailed characteristics of the studies included in this systematic review and meta-analysis are shown in Table 1.

**Table 1:** Main characteristics of the randomized controlled trials included in the systematic review and meta-analysis.

Study	Participants			Intervention				Outcome		Measuring instrument		Results
	Sample size (male and female), N	Characteristics	Sample size and age (years)	EG^a^	CG^b^	Duration and frequency						
El-Shamy and Alsharif [41], 2017	40 (27 and 13)	NL^c^: C5-C6; weight (kg)—EG: 21.65 (SD 1.76) and CG: 22.2 (SD 1.51); height (cm)—EG: 116.95 (SD 5.62) and CG: 119.41 (SD 5.41); affected side (RA^d^)—EG: 12 and CG: 13	EG: 20 (mean 6.35, SD 0.93); CG: 20 (mean 6.60, SD 1.05)	ASP^e^	CPT^f^	12 weeks; 3 sessions/week; 45 min/session	Functional activity; active ROM^g^; muscle strength		MSS^h^; goniometer; handheld dynamometer		Significant differences (EG or CG); SER^i^: *P*<.001, HN^j^: *P*<.001, HS^k^: *P*<.001, and HM^l^: *P*<.001 for functional activity; SABD^m^: *P*<.001 and SER: *P*<.001 for active ROM; SABD: *P*<.001 and SER: *P*<.001 for muscle strength	
Tarakci et al [43], 2019	19 (11 and 8)	NL: C5-C6, C5-C7, or C5-T1; weight (kg)—EG: 15.82 (SD 2.71) and CG: 16.85 (SD 2.74); height (cm)—ND^n^; affected side (RA)—EG: 8 and CG: 9	EG: 9 (mean 8.22, SD 2.58); CG: 10 (mean 8.30, SD 2.21)	LMC^o^	CPT	8 weeks; 3 sessions/week; 60 min/session	Functional activity; fine motor skills; muscle strength		DHI^p^, JTHFT^q^, and CHAQ^r^; 9HPT^s^; handheld dynamometer (HG^t^) or hydraulic pinch gauge (TG^u^)		Significant intragroup differences; DHI—EG: *P*=.006 and CG: *P*<.001 for functional activity; JTHFT—EG: *P*=.04 and CG: *P*=.001 for functional activity; EG: *P*=.02 and CG: *P*=.01 for fine motor skills; HG—EG: *P*=.007 and CG: *P*=.002 for muscle strength; TG—EG: *P*=.03 and CG: *P*=.003 for muscle strength	
Alsakhawi and Atya [40], 2020	45 (20 and 25)	NL: C5-C6, C5-C7, or C6-C7; weight (kg)—EG: 29.51 (SD 2.46) and CG: 30.25 (SD 2.90); height (cm)—EG: 129.67 (SD 1.67) and CG: 132 (SD 2.59); affected side (RA)—ND	EG: 22 (mean 6.55, SD 1.01); CG: 23 (mean 6.09, SD 0.95)	E-L ULE^v^	CPT	6 weeks; 3 sessions/week; 60 min/session	Active ROM; functional activity; muscle strength		Inclinometer; MSS; AMS^w^		Significant differences (EG or CG); SF^x^ (elbow flexion): *P*=.007, SF (elbow extension): *P*=.002, SABD: *P*=.002, and SER: *P*=.001 for active ROM; SABD: *P*=.01, SER: *P*=.03, HN: *P*=.001, HS: *P*=.004, and HM: *P*=.04 for functional activity; SF: *P*=.001, SABD: *P*=.001, and SER: *P*=.006 for muscle strength	
Yeves-Lite et al [11], 2020	12 (6 and 6)	NL: C5-C6 and C5-C7; weight (kg)—ND; height (cm)—ND; affected side (RA)—EG: 5 and CG: 3	EG: 6 (mean 8.50, SD 3.50); CG: 6 (mean 8.42, SD 3.40)	VRMT^y^ app	CMT^z^	4 weeks; 3 sessions/week; 20 min/session	Functional activity; quality of life		Children’s Hand-use Experience Questionnaire; Pediatric Quality of Life Inventory 4.0		Significant intragroup differences; independent tasks—EG: *P*=.02 for functional activity; use of the affected hand with grasp—EG: *P*=.04 for functional activity; children answers—EG: *P*=.04 for quality of life	
Karas et al [42], 2022	22 (11 and 11)	NL: C5-C6; weight (kg)—EG: 26.6 (SD 11.37) in female participants and 23.17 (SD 6.68) in male participant, CG: 24.33 (SD 7.03) in female participants and 28.6 (SD 5.03) in male participant; height (cm)—EG: 115.67 (SD 15.27) in female participants and 115.5 (SD 20.74) in male participants, CG: 116.0 (SD 13.19) in female participants and 106.5 (SD 10.71) in male participant; affected side (RA)—EG: 4 and CG: 7	EG: 11 (mean 7.33, SD 2.34 for female participants and mean 7.80, SD 4.09 for male participants); CG: 11 (mean 8.40, SD 2.19 for female participants and mean 6.67, SD 2.80 for male participants)	CG+ Wii Sports	CPT	6 weeks; 4 sessions/week; 40 min/session	Muscle strength; functional activity; active ROM		AMS; MSS; goniometer		Significant differences (EG or CG); SER: *P*=.001, shoulder internal rotation: *P*=.02, and forearm pronation: *P*=.03 for active ROM	

^a^EG: experimental group.

^b^CG: control group.

^c^NL: nerve lesion.

^d^RA: right arm.

^e^ASP: Armeo Spring Pediatric.

^f^CPT: conventional physical therapy.

^g^ROM: range of movement.

^h^MSS: Mallet scoring system.

^i^SER: shoulder external rotation.

^j^HN: hand to neck.

^k^HS: hand to spine.

^l^HM: hand to mouth.

^m^SABD: shoulder abduction.

^n^ND: not described.

^o^LMC: Leap Motion Controller.

^p^DHI: Duruoz Hand Index.

^q^JTHFT: Jebsen-Taylor Hand Function Test.

^r^CHAQ: Childhood Health Assessment Questionnaire.

^s^9HPT: Nine-Hole Peg Test.

^t^HG: hand grip.

^u^TG: tip grip.

^v^E-L ULE: E-LINK Upper Limb Exerciser.

^w^AMS: Active Movement Scale.

^x^SF: shoulder flexion.

^y^VRMT: virtual reality mirror therapy.

^z^CMT: conventional mirror therapy.

### Results of the Methodological Quality and Risk-of-Bias Assessment

The methodological quality of the RCTs included in this review was generally good (average total PEDro score 6.2, SD 0.84; range 5-7). In total, 80% (4/5) of the RCTs [40-43] had a good methodological quality, with a score of ≥6 points, as shown in Table 2.

**Figure 2:**
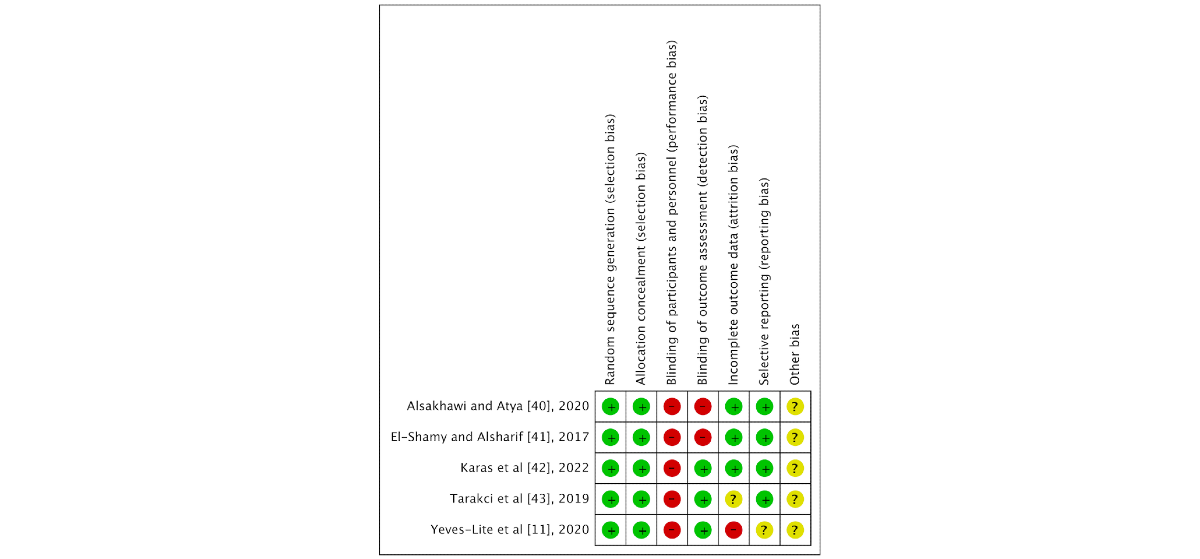
Risk of bias of the studies included in the systematic review. The green circle (+) indicates low risk of bias, the yellow circle (?) indicates unclear risk of bias, and the red circle (−) indicates high risk of bias [11,40-43].

**Figure 3:**
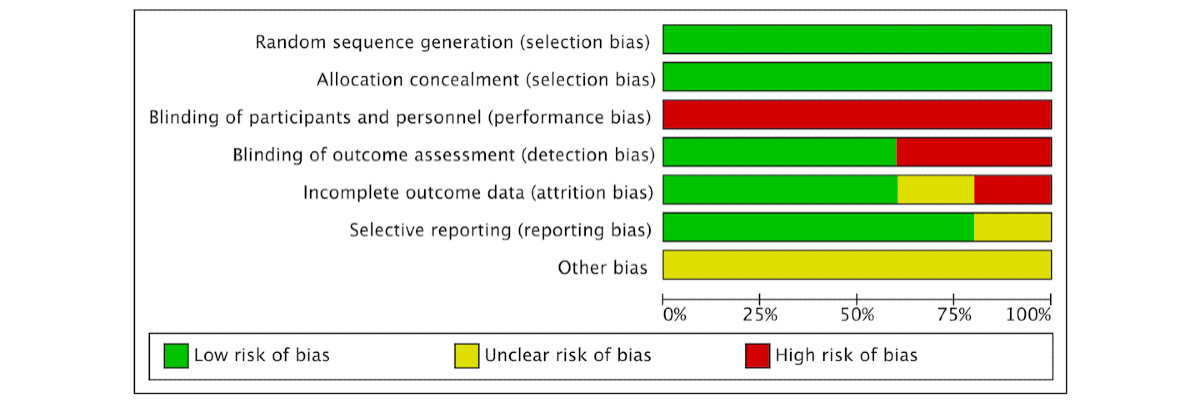
Overall risk of bias, with each category presented as percentages.

Figures 2 and 3 summarize the assessment of the risk of bias of the included RCTs both globally and individually for each study. When analyzed individually (Figure 2), the study by Karas et al [42] showed the lowest risk of bias, followed by the study by Tarakci et al [43]. The study by Yeves-Lite et al [11] showed the highest risk of bias. The “random sequence generation” and “allocation concealment” categories of selection bias had a 100% low risk of bias (Figure 3).

**Table 2:** Results obtained after the evaluation of methodological quality according to the PEDro scale.^a^

Study	C1^b^	C2^c^	C3^d^	C4^e^	C5^f^	C6^g^	C7^h^	C8^i^	C9^j^	C10^k^	C11^l^	Total score (out of 10)	Methodological quality
El-Shamy and Alsharif [41], 2017	1	1	1	1	0	0	0	1	0	1	1	6	Good
Tarakci et al [43], 2019	1	1	1	1	0	0	1	1	0	1	1	7	Good
Alsakhawi and Atya [40], 2020	1	1	1	1	0	0	0	1	0	1	1	6	Good
Yeves-Lite et al [11], 2020	1	1	0	1	0	0	1	0	0	1	1	5	Fair
Karas et al [42], 2022	1	1	1	1	0	0	1	1	0	1	1	7	Good

^a^*1* indicates that a study meets that criterion, and *0* indicates that the study does not meet the criterion or does not provide sufficient information to ensure it.

^b^C1: the choice criteria have been specified (not applied to calculate the score of the items on the PEDro scale).

^c^C2: participants were randomly assigned to groups.

^d^C3: treatment assignment was performed in a concealed manner.

^e^C4: groups had similar characteristics at baseline.

^f^C5: blinding of participants.

^g^C6: blinded therapists administering the treatment.

^h^C7: blinded assessors collecting measurements.

^i^C8: measures of at least 1 of the key outcomes were obtained from >85% of the participants initially assigned to the groups.

^j^C9: results were presented for all participants who received treatment or were assigned to the control group, or when this could not be done, data for at least 1 key outcome were analyzed on an “intention-to-treat” basis.

^k^C10: results of statistical comparisons between groups were reported for at least 1 key outcome.

^l^C11: the study provides point and variability measures for at least 1 key outcome.

### Synthesis of Results and Meta-Analysis

A total of 60% (3/5) of the RCTs [40-42] were included in the meta-analysis according to 3 different tests: MSS, AMS, and ROM. A high degree of heterogeneity (*I*^2^>50%) for each outcome (MSS, AMS, and ROM) was found. In addition, these tests were divided into different subtests: global abduction, global external rotation, hand to neck, hand to spine, and hand to mouth for the MSS (functional activity); shoulder flexion, shoulder abduction, and shoulder external rotation for the AMS (muscle strength); and shoulder abduction and shoulder external rotation for ROM.

**Figure 4:**
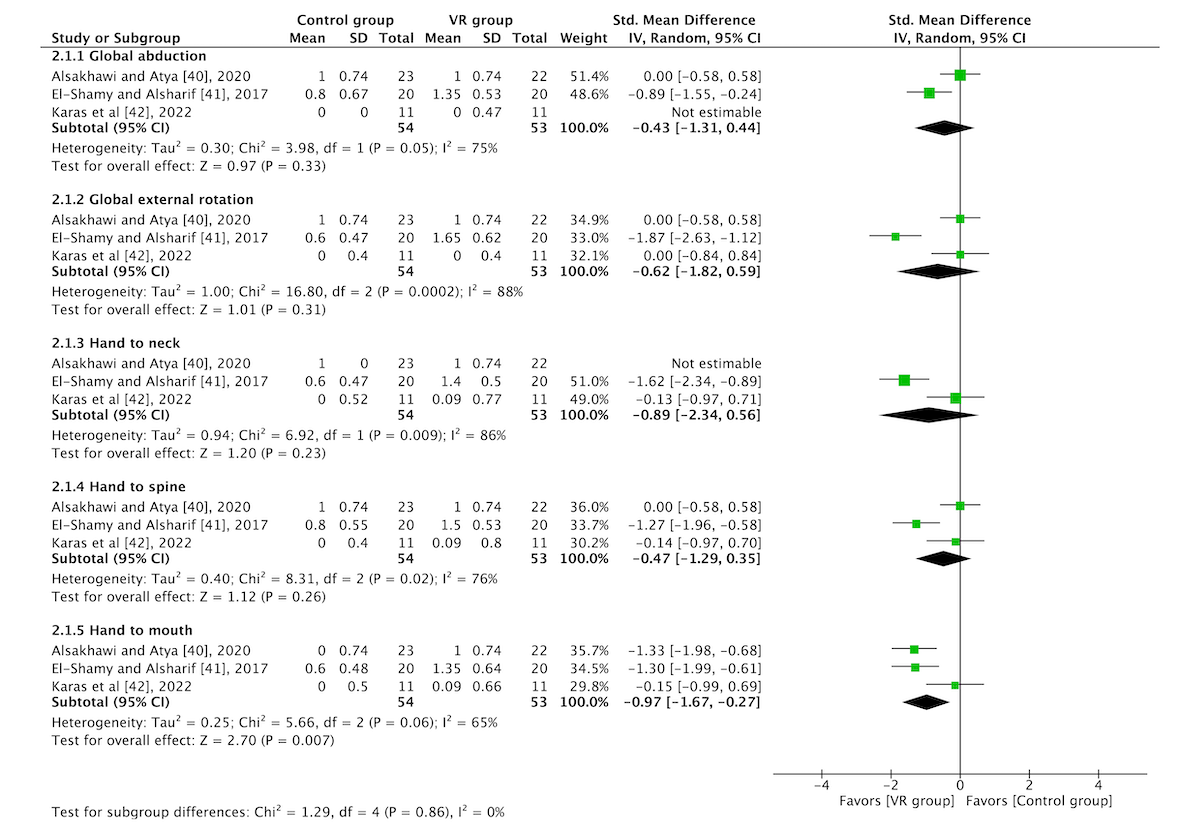
Forest plot for functional activity [40-42]. IV: inverse variance; VR: virtual reality.

In total, 60% (3/5) of the RCTs [40-42] were included in the MSS outcome within the functional activity analysis. The overall results of the subtest meta-analyses were not favorable except for the hand-to-mouth subtest (SMD=−0.97, 95% CI −1.67 to −0.27; *P*=.007). The global abduction (SMD=−0.43, 95% CI −1.31 to 0.44; *P*=.33), global external rotation (SMD=−0.62, 95% CI −1.82 to 0.59; *P*=.31), hand-to-neck (SMD=−0.89, 95% CI −2.34 to 0.56; *P*=.23), and hand-to-spine (SMD=−0.47, 95% CI −1.29 to 0.35; *P*=.26) subtests did not yield significant results. The study by El-Shamy and Alsharif [41] obtained the best results. The forest plots are shown in Figure 4.

**Figure 5:**
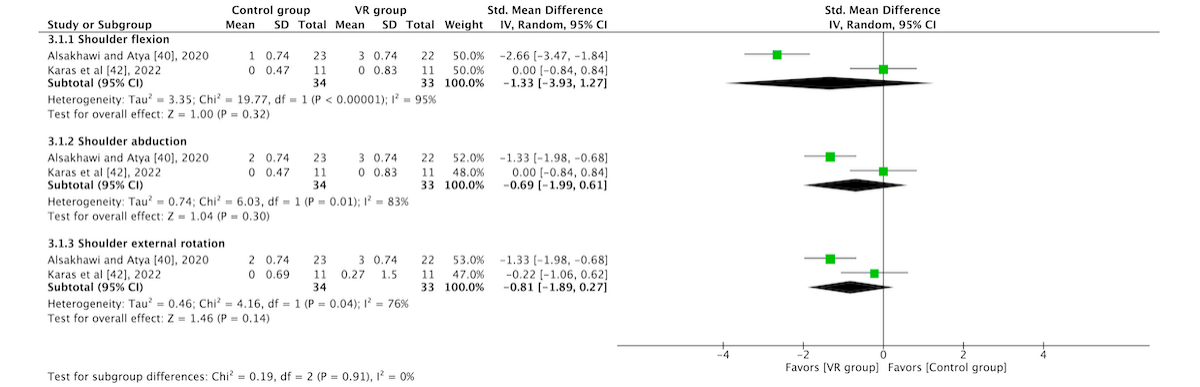
Forest plot for strength [40,42]. IV: inverse variance; VR: virtual reality.

In total, 40% (2/5) of the RCTs [40,42] were included in the AMS outcome within the muscle strength analysis. The overall results of the meta-analyses for the shoulder flexion (SMD=−1.33, 95% CI −3.93 to 1.27; *P*=.32), abduction (SMD=−0.69, 95% CI −1.99 to 0.61; *P*=.30), and external rotation (SMD=−0.81, 95% CI −1.89 to 0.27; *P*=.14) subtests were not significant. The study by Alsakhawi and Atya [40] obtained the best results. The forest plots are shown in Figure 5.

**Figure 6:**
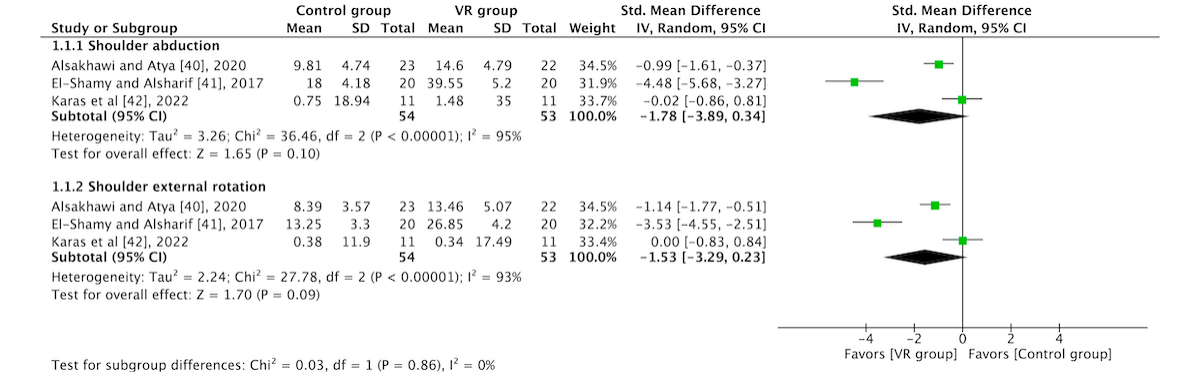
Forest plot for range of movement [40-42]. IV: inverse variance; VR: virtual reality.

In total, 60% (3/5) of the RCTs [40-42] were included in the ROM outcome. The overall results of the meta-analyses for the shoulder abduction (SMD=−1.78, 95% CI −3.89 to 0.34; *P*=.10) and external rotation (SMD=−1.53, 95% CI −3.29 to 0.23; *P*=.09) subtests were not significant. The study by El-Shamy and Alsharif [41] obtained the best results. The forest plots are shown in Figure 6.

Finally, the main meta-analysis findings are presented in Table 3.

**Table 3:** Main meta-analysis findings.

Outcome and subgroup		Included studies	Heterogeneity test	Statistical model	SMD^a^ (95% CI)	Overall effect	
						*z* score	*P* value
**Functional activity (MSS^b^)**							
	Global abduction	Alsakhawi and Atya [40] El-Shamy and Alsharif [41] Karas et al [42]	Heterogeneity	Random effects	−0.43 (−1.31 to 0.44)	0.97	.33
	Global external rotation	Alsakhawi and Atya [40] El-Shamy and Alsharif [41] Karas et al [42]	Heterogeneity	Random effects	−0.62 (−1.82 to 0.59)	1.01	.31
	Hand to neck	Alsakhawi and Atya [40] El-Shamy and Alsharif [41] Karas et al [42]	Heterogeneity	Random effects	−0.89 (−2.34 to 0.56)	1.20	.23
	Hand to spine	Alsakhawi and Atya [40] El-Shamy and Alsharif [41] Karas et al [42]	Heterogeneity	Random effects	−0.47 (−1.29 to 0.35)	1.12	.26
	Hand to mouth	Alsakhawi and Atya [40] El-Shamy and Alsharif [41] Karas et al [42]	Heterogeneity	Random effects	−0.97 (−1.67 to −0.27)	2.70	*.007* ^c^
**Strength (AMS^d^)**							
	Shoulder flexion	Alsakhawi and Atya [40] Karas et al [42]	Heterogeneity	Random effects	−1.33 (−3.93 to 1.27)	1.00	.32
	Shoulder abduction	Alsakhawi and Atya [40] Karas et al [42]	Heterogeneity	Random effects	−0.69 (−1.99 to 0.61)	1.04	.30
	Shoulder external rotation	Alsakhawi and Atya [40] Karas et al [42]	Heterogeneity	Random effects	−0.81 (−1.89 to 0.27)	1.46	.14
**ROM^e^**							
	Shoulder abduction	Alsakhawi and Atya [40] El-Shamy and Alsharif [41] Karas et al [42]	Heterogeneity	Random effects	−1.78 (−3.89 to 0.34)	1.65	.10
	Shoulder external rotation	Alsakhawi and Atya [40] El-Shamy and Alsharif [41] Karas et al [42]	Heterogeneity	Random effects	−1.53 (−3.29 to 0.23)	1.70	.09

^a^SMD: standardized mean difference.

^b^MSS: Mallet scoring system.

^c^Denotes significant results (*P*<.05).

^d^AMS: Active Movement Scale.

^e^ROM: range of movement.

## Discussion

### Principal Findings

The results of the 5 RCTs included in this systematic review suggest that VR interventions in patients with OBP were not superior to conventional therapy. To our knowledge, this is the first systematic review and meta-analysis to analyze the effects of VR in patients with OBP. In terms of the type of VR therapies, the studies used various technologies, including the Armeo Spring Pediatric [41], control sensors with video games [43], the E-LINK Upper Limb Exerciser [43], mirror therapy with VR [11], and video games with Nintendo Wii [42].

The methodological quality of the RCTs included in this review was generally good, with an average total PEDro score of 6.2 ranging from 5 to 7. A total of 80% (4/5) of the RCTs [40-43] had a good methodological quality, with a score of ≥6 points. The study by Karas et al [42] had the lowest risk of bias, followed by the study by Tarakci et al [43], indicating a higher quality of evidence. In contrast, the study by Yeves-Lite et al [11] had the highest risk of bias. Furthermore, it is worth noting that performance biases were the most common type of bias found. Therefore, it is important to keep in mind that these issues may affect the interpretation of the results. Nevertheless, RCTs in rehabilitation do not have double blinding as it is difficult or impossible to blind the patients and therapists who receive or deliver the interventions [44]. In that sense, some studies suggest that double blinding may not be necessary or valid for trials in real-life circumstances [45].

The results obtained by El-Shamy and Alsharif [41] showed that using Armeo Spring Pediatric was effective in improving functional activity. However, no significant results were found for the exercises performed using the Nintendo Wii [42] or the E-LINK exerciser [40] in this outcome except for the hand-to-mouth subtest in the latter. There could be several reasons why there are differences between them. Overall, the differences in the use of these devices could be due to a combination of factors related to the nature of the interventions, patient engagement and motivation, and the severity of OBP. In that way, the Nintendo Wii may be perceived as more of a leisure activity, and patients may not be as motivated to continue with their rehabilitation exercises. Furthermore, future research could focus on this aspect, analyzing whether patients with more severe OBP may require more specialized and intensive interventions such as the Armeo Spring Pediatric whereas patients with less severe OBP may benefit more from less specialized interventions such as the Nintendo Wii.

Concerning functional activity and shoulder ROM, measured using the MSS and goniometer, respectively, the best results for both outcomes were obtained by El-Shamy and Alsharif [41], who used Armeo Spring Pediatric and performed a longer-term intervention program (12 weeks). Therefore, we can suggest that the program duration could positively affect these outcomes. In addition, all RCTs conducted 3 to 4 sessions per week, which is usually the appropriate frequency for the treatment of OBP [46]. Nevertheless, the meta-analysis showed no significant results for either outcome except for the hand-to-mouth subtest of functional activity.

Regarding the ROM outcome, no significant results were found for shoulder abduction or shoulder external rotation. From a deeper analysis, it appears that the study conducted by Karas et al [42] obtained the lowest effects, influencing the overall results. Nevertheless, it is important to highlight that it had positive effects on shoulder flexion ROM, forearm pronation, and wrist flexion but not on the rest of the movements as these movements were not performed in the games. The lack of significant effects of VR therapy on these movements could be because these ranges were lower before treatment for the study participants relative to the control group and VR therapy enhances neuroplasticity by stimulating previously unused motor pathways and motor learning [47]. Moreover, the duration of each exercise session and follow-up can also affect ROM results. Longer exercise sessions may lead to greater improvements in ROM as the joints are moved through a greater range of motion for a longer period.

Finally, the meta-analysis showed no significant results for shoulder muscle strength. The results obtained by Alsakhawi and Atya [40] were higher than those obtained by Karas et al [42]. Although both studies used games as an intervention method, Alsakhawi and Atya [40] used an augmented biofeedback device designed to strengthen the upper limb muscles using different resistance levels within the games, so this aspect may have played a key role in the achievement of the results.

### Clinical Implications and Challenges

Although scientific literature supports the use of VR technologies for the rehabilitation of different diseases, providing several advantages such as enhancing patient motivation, providing direct feedback, and focusing the patient’s attention during the intervention [48], our systematic review and meta-analysis showed that VR was no more effective than conventional therapy for improving upper limb function, ROM, and strength in patients with OBP. In this way, the use of VR for upper limb rehabilitation in these patients is still in its first stages, and the quality of evidence is not enough to support its efficacy and strongly recommend its use. Nevertheless, considering the specific features of VR therapy, we can suggest that it could be used as a complement to conventional or other therapies to provide intensive repetition of meaningful task-related activities in an entertaining and challenging context to encourage children’s participation, which is necessary for the recovery of motor disorders of the central or peripheral nervous system [41,49].

Regarding the potential challenges of implementing VR-based interventions in clinical practice, it should be considered that patients may respond differently depending on their learning capacity to act in a virtual environment; their sensitivity or apprehensiveness; and the potential side effects that may occur during the intervention, such as cybersickness. In addition, clinicians should receive specific training on the proper use of VR technologies for therapeutic purposes [30]. Another challenge could be related to the high costs of integrating this therapy into clinical practice as it requires the purchase of high-quality hardware and software [31].

### Study Limitations and Recommendations for Future Research

This systematic review and meta-analysis presented several limitations that need to be considered. First, it should be noted that there is a lack of conclusive literature on whether VR treatments are superior to other types of therapy. Second, the small sample sizes in each study resulted in a lack of in-depth analysis, and the results were not evaluated in the long term. In addition, not all evaluations were performed by blinded professionals, which may have introduced bias to the study. Furthermore, the RCTs included in this systematic review did not test different doses to observe whether there was an improvement with higher doses. In addition, objective results such as the International Classification of Functioning, Disability, and Health (ICF) were not included in the RCTs. These limitations should be considered when interpreting the results of this systematic review and meta-analysis.

Concerning future research recommendations, in view of our results, some issues can be addressed by researchers. Future studies could investigate the effects of VR in the long term (eg, 6 months or a year) and with a larger sample size to determine whether the benefits are sustained. Furthermore, it could be investigated whether VR has different effects on different subgroups of patients (eg, according to age, gender, or severity of OBP), which could help tailor the treatment to the specific needs of each patient. Finally, in relation to the aforementioned limitation, future studies could investigate other outcomes, such as pain, anxiety, and depression, and include the ICF to enrich the results and increase their clinical utility.

This study has the potential to serve as a foundation for future RCTs. Through its findings, it highlights the need for further research that can provide a more nuanced and stratified analysis of the results. Such findings could prove invaluable in informing and guiding the development of more effective treatments and interventions in the medical field.

### Conclusions

This systematic review and meta-analysis aimed to evaluate the efficacy of VR therapy in patients with OBP. On the basis of the analysis of the effects on functional activity (except the hand-to-mouth subtest), muscle strength, and ROM, we cannot state that VR therapy is more effective than conventional therapies in upper limb rehabilitation in patients with OBP as no significant results were obtained. However, based on the VR therapy features, we can suggest that this therapy could be an adjunct to conventional therapies or other therapies to stimulate patient attention during the intervention, enhance patient motivation, and allow for the intensive repetition of meaningful task-related activities in an entertaining and challenging context.

In view of these results, this intervention is still in its first stages as the included RCTs presented several limitations that should be considered when interpreting the findings of this study. They had small sample sizes, limited long-term analysis, lack of testing of different doses, and absence of ICF-related outcomes; therefore, further research is needed to fully understand the potential of VR technologies as a therapeutic approach for patients with OBP. It is necessary to investigate the long-term effects of VR, explore individual differences to tailor treatment, and examine the potential differential effects on subgroups of patients. It is also recommended to incorporate other relevant outcomes, such as pain, anxiety, and depression, along with the ICF to enhance the clinical utility of the results. Such research could provide a stratified analysis of the benefits of VR therapy and, ultimately, inform the development of more effective treatments for patients with OBP. Overall, these findings highlight the need for continued research and development of these treatments.

## Appendix

Multimedia Appendix 1 PRISMA (Preferred Reporting Items for Systematic Reviews and Meta-Analyses) 2020 checklist.

Multimedia Appendix 2 Search strategy.

## Abbreviations

AMS: Active Movement Scale
ICF: International Classification of Functioning, Disability, and Health
MeSH: Medical Subject Headings
MSS: Mallet scoring system
OBP: obstetric brachial palsy
PICOS: population, intervention, comparison, outcome, and study
PRISMA: Preferred Reporting Items for Systematic Reviews and Meta-Analyses
RCT: randomized controlled trial
ROM: range of movement
SMD: standardized mean difference
VR: virtual reality
