# Supplementary material for: Virtual Reality for Upper Limb Rehabilitation in Patients With Obstetric Brachial Palsy: Systematic Review and Meta-Analysis of Randomized Controlled Trials
Source: J Med Internet Res. 2023 Jun 30;25:e47391. doi: 10.2196/47391 (PMC10365570; doi:10.2196/47391)
Supplement: Multimedia Appendix 2 [file jmir_v25i1e47391_app2.docx]

Multimedia Appendix 2. Search strategy.

| DATABASES | | NUMBER  OF  ARTICLES | SEARCH  TERMS | |
| --- | --- | --- | --- | --- |
| PubMed | | 19 | ("Neonatal Brachial Plexus Palsy"[Mesh] OR "Brachial Plexus Neuropathies"[Mesh] OR "Brachial Plexus"[Mesh] OR "brachial plexus" OR "brachial plexus block*" OR "brachial plexus blockade*" OR "brachial plexus neuritis" OR "brachial neuritis" OR "amyotrophic neuralgia" OR "neuralgic amyotrophy" OR "brachial neuralgia" OR "cervicobrachial neuralgia" OR "brachial plexopathy" OR "brachial plexus neuropathy" OR "brachial plexus neuropathies" OR "brachial plexus disorder*" OR "brachial plexus disease*" OR "klumpke paralysis" OR "erb paralysis" OR "klumpke palsy" OR "erb palsy" OR "neonatal brachial plexus palsy" OR "obstetrical brachial plexus palsy" OR "obstetrical brachial plexus lesion") AND ("Virtual Reality"[Mesh] OR "Virtual Reality Exposure Therapy"[Mesh] OR "Exergaming"[Mesh] OR "virtual reality exposure therapy" OR "virtual reality" OR "augmented reality" OR "virtual system*" OR "video game*" OR "videogame*" OR "exergaming" OR "exergame*" OR "commercial game*" OR "play-based" OR "game-based") | |
| Scopus | | 48 | TITLE-ABS-KEY ( (“brachial plexus” OR “brachial plexus block*” OR “brachial plexus blockade*” OR “brachial plexus neuritis” OR “brachial neuritis” OR “amyotrophic neuralgia” OR “neuralgic amyotrophy” OR “brachial neuralgia” OR “cervicobrachial neuralgia” OR “brachial plexopathy” OR “brachial plexus neuropathy” OR “brachial plexus neuropathies” OR “brachial plexus disorder*” OR “brachial plexus disease*” OR  “klumpke paralysis” OR “erb paralysis” OR “klumpke palsy” OR “erb palsy” OR “neonatal brachial plexus palsy” OR “obstetrical brachial plexus palsy” OR “obstetrical brachial plexus lesion”) AND ("virtual reality exposure therapy" OR "virtual reality" OR "augmented reality" OR "virtual system*" OR "video game*" OR "videogame*" OR "exergaming" OR "exergame*" OR "commercial game*" OR "play-based" OR "game-based")) | |
| WOS  All Databases | | 31 | TS=((“brachial plexus” OR “brachial plexus block*” OR “brachial plexus blockade*” OR “brachial plexus neuritis” OR “brachial neuritis” OR “amyotrophic neuralgia” OR “neuralgic amyotrophy” OR “brachial neuralgia” OR “cervicobrachial neuralgia” OR “brachial plexopathy” OR “brachial plexus neuropathy” OR “brachial plexus neuropathies” OR “brachial plexus disorder*” OR “brachial plexus disease*” OR  “klumpke paralysis” OR “erb paralysis” OR “klumpke palsy” OR “erb palsy” OR “neonatal brachial plexus palsy” OR “obstetrical brachial plexus palsy” OR “obstetrical brachial plexus lesion”) AND ("virtual reality exposure therapy" OR "virtual reality" OR "augmented reality" OR "virtual system*" OR "video game*" OR "videogame*" OR "exergaming" OR "exergame*" OR "commercial game*" OR "play-based" OR "game-based")) | |
| Cochrane | | 13 | (“brachial plexus” OR “brachial plexus block*” OR “brachial plexus blockade*” OR “brachial plexus neuritis” OR “brachial neuritis” OR “amyotrophic neuralgia” OR “neuralgic amyotrophy” OR “brachial neuralgia” OR “cervicobrachial neuralgia” OR “brachial plexopathy” OR “brachial plexus neuropathy” OR “brachial plexus neuropathies” OR “brachial plexus disorder*” OR “brachial plexus disease*” OR  “klumpke paralysis” OR “erb paralysis” OR “klumpke palsy” OR “erb palsy” OR “neonatal brachial plexus palsy” OR “obstetrical brachial plexus palsy” OR “obstetrical brachial plexus lesion”) AND ("virtual reality exposure therapy" OR "virtual reality" OR "augmented reality" OR "virtual system*" OR "video game*" OR "videogame*" OR "exergaming" OR "exergame*" OR "commercial game*" OR "play-based" OR "game-based") in Title Abstract Keyword | |
| ProQuest | | 29 | (“brachial plexus” OR “brachial plexus block*” OR “brachial plexus blockade*” OR “brachial plexus neuritis” OR “brachial neuritis” OR “amyotrophic neuralgia” OR “neuralgic amyotrophy” OR “brachial neuralgia” OR “cervicobrachial neuralgia” OR “brachial plexopathy” OR “brachial plexus neuropathy” OR “brachial plexus neuropathies” OR “brachial plexus disorder*” OR “brachial plexus disease*” OR  “klumpke paralysis” OR “erb paralysis” OR “klumpke palsy” OR “erb palsy” OR “neonatal brachial plexus palsy” OR “obstetrical brachial plexus palsy” OR “obstetrical brachial plexus lesion”) AND ("virtual reality exposure therapy" OR "virtual reality" OR "augmented reality" OR "virtual system*" OR "video game*" OR "videogame*" OR "exergaming" OR "exergame*" OR "commercial game*" OR "play-based" OR "game-based") | |
| CINAHL  Complete | | 8 | (“brachial plexus” OR “brachial plexus block*” OR “brachial plexus blockade*” OR “brachial plexus neuritis” OR “brachial neuritis” OR “amyotrophic neuralgia” OR “neuralgic amyotrophy” OR “brachial neuralgia” OR “cervicobrachial neuralgia” OR “brachial plexopathy” OR “brachial plexus neuropathy” OR “brachial plexus neuropathies” OR “brachial plexus disorder*” OR “brachial plexus disease*” OR  “klumpke paralysis” OR “erb paralysis” OR “klumpke palsy” OR “erb palsy” OR “neonatal brachial plexus palsy” OR “obstetrical brachial plexus palsy” OR “obstetrical brachial plexus lesion”) AND ("virtual reality exposure therapy" OR "virtual reality" OR "augmented reality" OR "virtual system*" OR "video game*" OR "videogame*" OR "exergaming" OR "exergame*" OR "commercial game*" OR "play-based" OR "game-based") | |
| PEDro | Search #1 | 33 | “brachial plexus” |  |
|  | Search #2 | 13 | “brachial palsy” |  |
